# Preparation of chitosan nanoparticle containing recombinant CD44v antigen and evaluation of its immunization capacity against breast cancer in BALB/c mice

**DOI:** 10.1186/s12885-023-10614-x

**Published:** 2023-02-09

**Authors:** Elaheh Gheybi, Ahmad Asoodeh, Jafar Amani

**Affiliations:** 1 Department of Chemistry, Faculty of Science, Ferdowsi University of Mashhad, Mashhad, Iran; 2 Cellular and Molecular Research Group, Institute of Biotechnology, Ferdowsi University of Mashhad, Mashhad, Iran; 3 Applied Microbiology Research Center, Systems Biology and Poisonings Institute, Baqiyatallah University of Medical Sciences, Tehran, Iran

**Keywords:** Breast cancer, Chitosan, Nanoparticle, Recombinant protein, CD44v

## Abstract

**Objective(s):**

Breast tumors show heterogeneity containing cancer stem cells as a small subpopulation of a tumor mass. CD44 as a cancer stem cells antigen is abnormally expressed by carcinomas of epithelial origin. Also, overexpression of CD44 variable isoforms (CD44v) is associated with malignancy in breast cancer. In the present research, our objective was to evaluate the immunogenicity of prepared nanoparticles containing a novel recombinant CD44v (rCD44v) protein in the mouse model.

**Materials and methods:**

CD44 gene was expressed in *E. coli* BL21 DE3 using the pET28a-CD44 vector. The expressed rCD44v protein was purified, encapsulated into the chitosan nanoparticles, and administered to BALB/c mice. ELISA was used to evaluate the immunoglobulin levels of immunized animals. For challenge experiment, 2 × 10^6^ 4T1-CD44 tumor cells were injected subcutaneously in mice, and tumor size, necrosis, and metastases were measured. Finally, cell proliferation assay, cytokines assay, and neutralization assay of the mouse anti-rCD44v on the human breast cancer cell line were examined.

**Results:**

The measured size of chitosan-rCD44v nanoparticles was 146.5 nm. Recombinant CD44v encapsulated by chitosan nanoparticles increases immunological responses via the adjuvant nature of chitosan nanoparticles. In the immunized mice, IgG and IgA titers were significantly increased. Tumor growth in injection and nano-injection test groups compared with the mice control groups displayed a significant reduction (*P* < 0.05). A high amount of splenocytes secreting IFNγ and IL-17 was seen in immunized mice with rCD44v (*P* < 0.05). Furthermore, a smaller size of lung metastases compared to the control mice groups was detected.

**Conclusion:**

The encapsulated rCD44v within the chitosan nanoparticles induced a significant immune response in mice and can establish significant protection against breast cancer. Therefore, it can be considered a vaccine candidate for breast cancer therapeutic modalities.

**Supplementary Information:**

The online version contains supplementary material available at 10.1186/s12885-023-10614-x.

## Highlights

rCD44v encapsulated by chitosan nanoparticles increases immunological responses.

rCD44v increased IgG and IgA titers and some cytokines in the immunized mice.

The rCD44v protein has potential features to be used as a vaccine for breast cancer.

**Supplementary Information:**

The online version contains supplementary material available at 10.1186/s12885-023-10614-x.

## Introduction

Breast cancer, as the main cause of cancer mortality among woman, show nearly 40% recurrence in metastatic disease, although the diagnosis and treatment of breast cancer are progressed through the past decades. [1]. There is different evidence confirming the existence of a stem cell-like population of cells probably responsible for the tumor’s origin and its maintenance. Although the number of these cells is very low in comparison with differentiated tumor cells in tumor mass, these cells are essential for tumor cell progression and development, as well as resistance to therapeutic modalities. Traditional therapies against breast cancer such as surgery, chemotherapy, and radiation therapy effectively debulk some tumors but often are unsuccessful for long-term usage, probably because they fail to destroy the stem cell-like subpopulation in tumors. Therefore, novel therapeutic modalities target these stem cells resembling populations to improve both primary and metastatic tumors. At the center of this era, cancer stem cell antigens meaningfully provide an interesting target for immunotherapy of cancer [2–4].

Immunotherapy, as a renewed interest in cancer treatment, stimulates immune cells to recognize and eradicate tumors. A variety of solid tumor vaccines have been evaluated in cancer immunotherapy. Increased knowledge about the immune system and tumor-associated antigens (TAAs, as targets for immunotherapy) bolstered the interest in these vaccines. In breast cancer, like other cancers, the immune system is impaired by tumors during cancer progression, Therefore, if the cancer antigens were introduced artificially to the immune system, it can recognize and eliminate cancer cells.

So, targeting the breast cancer stem cells (BCSCs) antigens could potentially eliminate this cancer [1–3].

The CD44, as a key antigen of cancer stem cells (CSCs), is a transmembrane glycoprotein receptor. Despite the existence of a single gene for CD44, different CD44 variants (CD44v) are expressed in cells, through altered splicing and/or glycosylation modification [5]. Since this molecule plays an essential role in different activities of cancer cells new hopes raised for immunotherapy to target CD44-expressing malignant cells [6].

Malak Hassn Mesrati et al. reviewed the different CD44 isoform structures and their functional roles in tumourigenesis and discussed the regulation of CD44 expression, as well as the CD44 signaling pathway involved in cancer progression and development. They also addressed the clinical significance and prognostic value of CD44 and its potential as a therapeutic target in cancer[7]. In breast tumors, the expression of different CD44 variants is associated with the aggressiveness of the cancer cells [4].

Due to abundance, using anti-CD44 agents which can recognize a shared epitope by all different CD44 variants would be more practical for CD44 targeting, compared to those agents which recognize a specific variant epitope. Thus, the CD44-negative cells with normal biology will be safe from vaccine off-targeting [3]. Therefore, vaccination against CD44v may inhibit tumor recurrence in cancer patients. Since breast cancer is widely abundant, there is an urgent to develop an effective vaccine for the disease [8].

A focused interest is obvious in subunit vaccines due to their high immunogenicity and low risk in cancer vaccination [9]. Furthermore, to escape vaccines from enzymatic degradation, encapsulation of protein using nanoparticles such as chitosan is useful not only for vaccine protection but also for its transportation into the epithelial tissue [10, 11].

Chitosan, as a natural polymer, presents some valuable advantages. While these non-toxic nanoparticles are effectively able to adhere to the intestinal epithelium, they present also biocompatibility, and antimicrobial effects [9]. Furthermore, these nano-size particles can stimulate the immune system with prolonged exposure to the vaccine in the environment, leading to gradually increased cytokines, and the functionality of the immune system, as well as improvement of vaccine effects [12–14] on the mucosal and cellular immune responses [15, 16].

In the current study, a short common section of variable area exons in the extracellular domain of human CD44 was chosen in this investigation and employed the recombinant CD44v (rCD44v) as a recombinant antigen [17], was nanoparticulated by chitosan. The immunogenicity capacity of mixing candidates, as a cancer vaccine, was evaluated in BALB/c mice.

## Materials and methods

### Materials

Chemical reagents, kits, and molecular markers from Merck (Germany), Gobiz (Korea), Sinaclon (Iran), Qiagen (Germany), G-Sepharose, anti-mouse IgG and IgA antibodies, and chitosan with an average molecular weight from Sigma-Aldrich (Germany). Sodium tripolyphosphate TPP was prepared (Scharlau, Spain). In addition, Dimethylthiazol-2-yl)-2, 5-diphenyltetrazolium bromide (MTT), 3-(4,5-, fetal bovine serum (FBS), Dulbecco’s modified Eagle’s medium (DMEM), penicillin-streptomycin and trypsin-EDTA were provided by Biosera (France).

### Cell line and culture condition

4T1 and MCF7 CD44 expressing breast cancer cell lines and the human embryonic kidney normal cell line HEK293 were purchased from the cell bank of Pasture Institute (Tehran, Iran). The cells were cultured in DMEM medium containing 10% FBS and 1% penicillin-G/streptomycin in a humidified incubator under 5% CO_2_ at 37 °C.

### Construct design and preparation of recombinant CD44v protein

The sequence of the antigenic variable part of the human CD44 (CD44v) extracellular domain (441–540 aa) was selected. This domain is representing three-dimensional epitopes. The constructed sequence was synthesized as a clone into the pET28a vector by General Biosystems, *Inc* (Durham, USA). The optimized sequence of the recombinant gene was published in GenBank (MT396985).

*E. coli* BL21(DE3) was transformed with the pET28a vector containing the CD44v gene (pET28a-CD44v) and confirmed as previously described [17]. The IMAC (Immobilized Metal Affinity Chromatography) was used to purify the recombinant 6 His-tagged CD44v antigens using Ni–NTA agarose (Qiagen) under native conditions. The isolated recombinant protein was verified using SDS-PAGE and Western blotting assays [17].

### Preparation of nanoparticles including rCD44v protein

Chitosan nanoparticles were used for encapsulation of the rCD44v protein by an ionic cross-linking method [18]. In a final volume of 25 mL of 2% acetic acid, 50 mg chitosan was dissolved and passed through a 0.45-micron filter. 500 µg of rCD44v antigen was added drop-wise into 7.5 mL chitosan solution (2 mg/mL) for 10 min in an adjusted pH 5.5 by NaOH (1 M). 5 mL of tripolyphosphate TPP (1 mg/mL) was added gradually to the mixture of chitosan-purified antigen at room temperature for 1 h. Nanoparticle aggregation was inhibited through sonication. A combination of chitosan nanoparticles with distilled water (equivalent to the used rCD44v amount) was encapsulated and used as the negative control, according to the protocol. Following aliquots of 1 mL of the mixture were centrifuged for 15 min at 13,000 rpm. The pellet was resuspended in 100 µL of phosphate buffer saline (PBS) and stored at -20 °C for further characterization.

### Size and the surface charge determination of nanoparticle

To measure the particle size, polydispersity index (PDI), and zeta potential of rCD44v nanoparticles, dynamic light scattering (DLS) and laser doppler electrophoresis using Zeta sizer (Nano-ZS, Malvern, UK) were used at a wavelength of 633 nm and 25 ºC. Zeta potential is measured in millivolts (mV), and it seems a logical term in colloidal dispersions for electrokinetic potential. In addition, it is a parameter for the physical stability of chitosan nanoparticles. The higher electrostatic repulsion among the particles is respected to the higher stability [19]. Before measurement samples were dispersed in distilled water. Three replicates were measured and values were presented as mean ± standard deviation (SD).

### Encapsulation efficiency

To determine the loading percentage, the mixture of chitosan- rCD44v nanoparticles were centrifuged (for 15 min at13,000 rpm) and the concentration of recombinant protein rCD44v in the supernatant was estimated via Bradford protein assay [20]. The efficiency of encapsulation was measured based on the following formula [21]:

$$\begin{array}{l}{\rm{Entrapment}}\,{\rm{Efficiency}}\left( {{\rm{EE}}\% } \right) = \\\,\,\,\,\,\,\,\,\frac{{({\rm{Totalantigen - antigeninsupernatant}})}}{{{\rm{Totalantigen}}}} \times 100\end{array}$$

### Antigen-releasing from nanoparticles

Some samples of chitosan-rCD44v nanoparticles were prepared in 10 mL of PBS (pH 7.4) and incubated in a shaker incubator at 100 rpm and 37 ºC. In an interval of 0, 1, 3, 12, 24, 48, 72, 96, and 120 h, 1 mL of the suspension was taken and centrifuged and a 100 µL sample was withdrawn. Bradford assay was used to determine the quantity of the released recombinant antigen from nanoparticles. A sample made from unloaded chitosan nanoparticles resuspended in PBS was used as a blank [22].

### Immunogenicity in an animal model

In the prophylactic immunization experiment of rCD44v, forty-four females inbred 6- to 7-week-old BALB/c mice (Pasteur Institute, Tehran, Iran) were divided into four tests groups oral, oral-injection, injection, and nanoparticle-injection (T1, T2, T3, T4) and two control groups injection and oral with PBS and chitosan nanoparticles without antigen (C1, C2), respectively. In the oral group (T1), each mouse was immunized directly by oral gavage administration of four doses of rCD44v nanoparticles (100 µg). For oral-injection mice (T2), three doses with the same amount were administered orally, and one dose of pure rCD44v protein (5 µg) was injected intraperitoneally for the last period. In the injection group (T3), the mice were injected in four doses (the first three doses were subcutaneous with 20 µg rCD44v protein, and the last one was intraperitoneal with 5 µg pure rCD44v). In the injection group, complete Freund,s adjuvant (CFA) was used for the first time with the same amount of antigens and incomplete Freund adjuvant (IFA) for the second and third times. While the CFA was applied to induce both humoral and cellular immune responses, IFA was used to enhance the humoral immune response after the first injection [23, 24]. In this group, the last injection was administered without an adjuvant. In the nanoparticle-injection group (T4), the encapsulated rCD44v protein (30 µg) was injected in four doses (three subcutaneously, and the last one intraperitoneally with 5 µg pure rCD44v). PBS and non-loaded chitosan nanoparticles were administered gavage for the injection (C1) and oral control (C2) groups, respectively. Administrations were conducted at intervals of two weeks.

Table 1 defines all the applied mice groups. The blood and fresh feces samples of each group) were added to 500 µL PBS. After homogenization, the liquid supernatant of the fecal was separated after centrifugation (4 °C, 6,000 rpm, 10 min) with serum samples kept at -20 °C for further analyses.

**Table 1:** Immunization administered to the groups of mice

Experimental group	Route of administration	Administrated formulation	Immunized Schedule		Adjuvant	Antigen dose (µg/mice)
			Step	Day		
**T1,Oral** **n = 8**	gavage	Nanoparticle rCD44v	1st	0	-	100
			2nd	14	-	100
			3rd	28	-	100
			4th	42	-	100
**T2, Oral- Injection** **n-=8**	gavage	Nanoparticle rCD44v	1st	0	-	100
			2nd	14	-	100
			3rd	28	-	100
	intrapersonal	Pure rCD44v	4th	42	-	5
**T3, Injection** **n = 8**	subcutaneous	rCD44v with adjuvant	1st	0	CFA	20
			2nd	14	IFA	20
			3rd	28	IFA	20
	intrapersonal	Pure rCD44v	4th	42	-	5
**T4, Nano- Injection** **n = 8**	subcutaneous	Nanoparticle rCD44v	1 st	0	-	30
			2nd	14	-	30
			3rd	28	-	30
	intrapersonal	Pure rCD44v	4th	42	-	5
**C1** **n = 6**	subcutaneous	PBS or Chitosan Without Antigen	1st	0	-	-
			2nd	14	-	-
			3rd	28	-	-
			4th	42	-	-
**C2** **n = 6**	gavage	Chitosan Without antigen	1st	0	-	-
			2nd	14	-	-
			3rd	28	-	-
			4th	42	-	-

### Determining the serum IgG antibody against rCD44v

Specific antibody responses were examined by an indirect -ELISA. The purified rCD44v protein (1 µg/ well) was coated in 96-well plates with coating buffer (Na_2_CO_3_, NaHCO_3_, pH 9.8) (Nunc, Denmark). All incubations were accomplished for 16 h at 4 °C. The plates were washed away with PBST buffer (PBS containing 0.05% Tween-20) three times, and 100 µL of 5% BSA was used to block the non-specific sites for 1 h at 37 °C and washed away PBST. The wells were incubated with a serially diluted serum of immunized mice as the primary antibody from 1:50 to 1:12,800 in PBST in triplicate at 100 µL/well and 37 °C for 1 h. After washing, the antibody detection was carried out with PBST and HRP anti-mouse IgG (1/2000 in PBST) (Sigma) as a conjugated secondary antibody, followed by the addition of substrate O-phenylenediamine (OPD) (Sigma) and placed in the dark place at room temperature for 15 min. The reaction was stopped with H_2_SO_4_ (2.5 M). ELISA reader (BioTek, ELX800, USA) was utilized to measure the absorbance at 492 nm.

### Determination of serum and fecal IgA antibody responses to rCD44v

1:5 to 1:640 serum dilutions were considered to define the IgA titer in all groups, using anti-IgA HRP conjugate (1/1000 in PBST) (Sigma). The secretory IgA in fecal samples was also evaluated. After centrifugation, the liquid supernatant of the fecal was diluted from 1:5 to 1:640 and examined by indirect ELISA.

### IgG antibody purification

Affinity chromatography was used to purify the mice-specific antibodies by using G-Sepharose column (Sigma). Briefly, after 1:3 ratio serum dilution with Binding Buffer or PBS (pH 7.2). Washing was conducted using 100 mM glycine (pH 3), and the eluted fractions were instantly neutralized by adding 100 µL of 1 M Tris-base (pH 8.5) per mL of the eluate. Then, the eluted antibodies were dialyzed against PBS (pH 7.2) overnight at 4 °C. ELISA and Bradford assays were applied to assess the reactivity and concentration of the purified antibodies, respectively.

### Neutralization assay of the mouse anti-rCD44v antibody

The cytotoxic activity of pure the mouse anti-rCD44v antibody was evaluated using an MTT assay as a colorimetric analysis. To this end, a density of 3 × 10^4^ of MCF7 and HEK293 cells were seeded per well in a 96-well plate and subjected to different concentrations of the pure mouse anti-rCD44v antibody (2 to 0.125 µg) at 37 °C for 24- and 48-hours incubation. The untreated cells were defined as the control. 100 µL of MTT solution (0.5 mg/mL) was poured into each well and incubated for 4 h. The culture supernatant was replaced with 100 µL DMSO to dissolve formazan crystals. The optical intensity (OD) of each well was read at 570 nm using an ELISA reader (BioTek, ELX800, USA). To determine cell viability the following equation was used [25]. The values of the half-maximal (50%) inhibitory concentration of cell proliferation (IC_50_) were measured using GraphPad Prism8 software.

$$\begin{array}{l}{\rm{Cell}}\,{\rm{viability}}\left( \% \right) = \\\frac{{({\rm{Absorbance}}\,{\rm{of}}\,{\rm{treated}}\,{\rm{cells - Absorbance}}\,{\rm{of}}\,{\rm{background}})}}{{({\rm{Absorbance}}\,{\rm{of}}\,{\rm{untreated}}\,{\rm{cells - Absorbance}}\,{\rm{of}}\,{\rm{background}})}} \times 100\end{array}$$

### Challenges investigation in immunized mice

For tumor formation, 2 × 10^6^ 4T1-CD44 tumor cells in 100 µL of PBS were injected subcutaneously four weeks after the last immunization into the right flanks of mice [26]. The general condition of the mice was monitored and the tumor growth was measured using a digital caliper every day. The following formula was used to calculate each tumor volume in mm^3^[27] :

$${\rm{V}} = {\rm{ }}0.5{\rm{ }} \times {\rm{ D}} \times {{\rm{d}}^2}$$

Where, V: volume; D: longitudinal diameter; d: latitudinal diameter.

### Cellular immune responses in rCD44v immunized mice

The mice spleens were collected under aseptic conditions and cut into small pieces, rinsed twice with PBS, and minced using forceps and a scalpel. To make a single-cell suspension, a 100 μm stainless steel mesh was used. Erythrocyte lysis was performed using ACK lysis buffer (NH_4_Cl, KHCO_3_, and Na_2_-EDTA) at room temperature. 2 × 10^5^ cells/well of prepared splenocytes were seeded in two 96-well plates, to perform cell proliferation and cytokine assays. Co-stimulated with purified rCD44v (100 ng/well) and incubated at 37 °C and 5% CO_2_ pressure. A medium alone (unstimulated control) was kept as control. To measure cell proliferation, 100 µL of MTT solution (0.5 mg/mL) was added to each well after 72 h post-stimulation, and incubated for 4 h, followed by adding DMSO. Then, the OD was measured by a BioTek microplate reader at 570 nm. The following formula was used to calculate the stimulation index (SI): mean OD of test cells / mean OD of control cells × 100 and the splenocytes were used without proximity to antigen rCD44v [28, 29].

For cytokine assay, the supernatants of the cells were collected and tested for cytokines interferon γ (IFN-γ), interleukin-17 (IL-17), and interleukin-4 (IL-4) by sandwich-based ELISA kits (R&D, Minneapolis, MN, USA) according to the manufacturer’s instructions. All experiments were carried out in triplicates [30].

### Histopathology experiments

The histopathology of tumors and some organs of mice were analyzed. Following tissue fixation in 10% formalin embedding in paraffin, three-µm-thick slices were prepared and mounted on glass slides. Paraffin was removed and the slides were stained with hematoxylin and eosin (H&E). Necrosis and metastases were analyzed using light microscopy (Olympus BX51, Tokyo, Japan) [31].

### Statistical analysis

Prism 8 (GraphPad Software, La Jolla, CA) software was used for statistical comparison. The independent t-test was applied for two-group comparison or one-way ANOVA (comparison of multiple means). The results were expressed as mean ± standard deviation (SD) of three independent experiments. The data with *P* ≤ 0.05 were considered significant.

## Results

### Confirmation of construct

Confirmation of the PET28a-cd44v construct was performed using PCR and recombinant CD44v protein with appropriate molecular size was detected in SDS-PAGE and confirmed immunologically through anti-His-tag antibodies using Western blot [17].

### Physical properties of chitosan nanoparticles containing rCD44v antigen

**Fig. 1:**
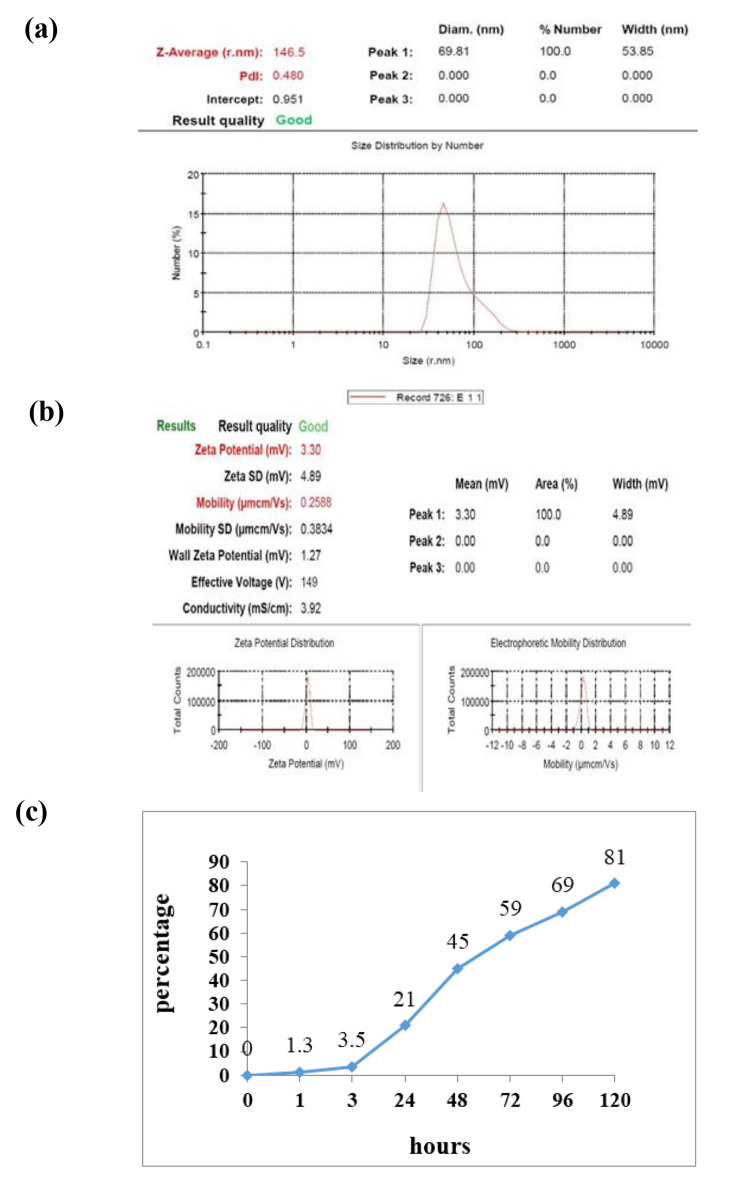
Measurement of the physical property of nanoparticles with recombinant protein. (**a**) Size distribution measured of rCD44v loaded with chitosan by Zetasizer. (**b**) Zeta potential and Mobility of chitosan nanoparticles containing rCD44v. (**c**) Graph of in vitro antigens release of nanoparticle rCD44v base on chitosan in PBS buffer. The mean ± SD of triplicate determinations is shown

Figure 1a describes the particle size and Zeta potential of rCD44v nanoparticles. The size of rCD44v nanoparticles was nearly 146.5 ± 6.42 nm with a PDI of about 0.480 ± 0.07, which indicates a homogenous size distribution. The value of + 3.3 ± 0.16 mV was measured as the surface charge of rCD44v nanoparticles indicating good mobility and its better adhesion to intestinal mucosal cells for the chitosan nanoparticles (Fig. 1b). The encapsulation efficiency of rCD44v in chitosan was 93%. This confirmed that the majority of the rCD44v antigen was loaded into the chitosan nanoparticles. Figure 1c depicts the release of antigens under in vitro conditions. During 120 h, 81% of rCD44v antigens were released in PBS buffer from chitosan nanoparticles.

### Immunological response analysis (IgG and IgA antibody) against rCD44v antigen

**Fig. 2:**
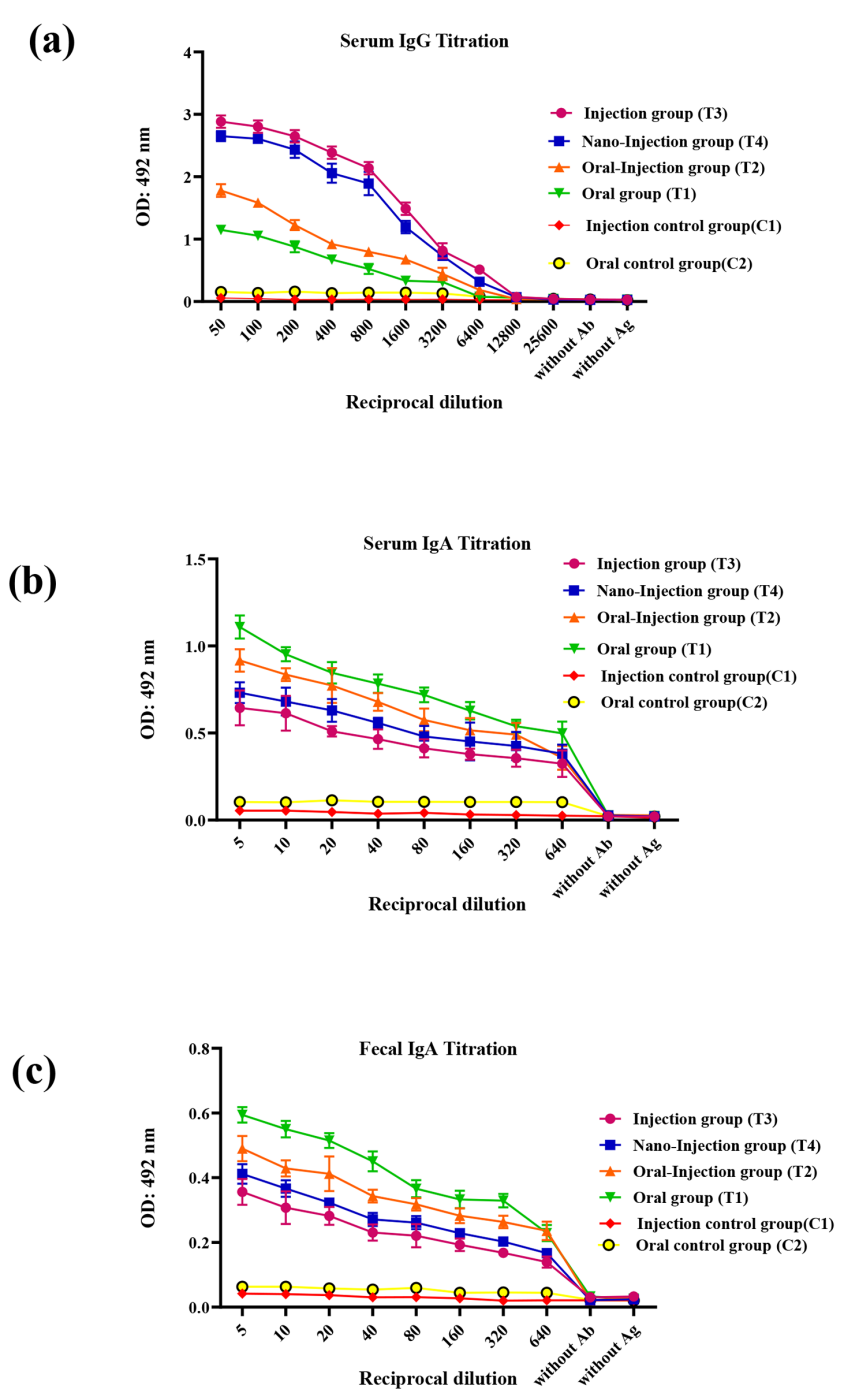
Specific serum and fecal antibody titer against recombinant CD44v in all groups. (**a**) Serum IgG titers the groups. (**b**) Serum IgA titers among the groups. (**c**) Fecal IgA titers among the groups. The mean ± SD of triplicate determinations is shown

The extent of the specific IgG and IgA antibodies against rCD44v proteins was measured using ELISA. The titers of serum anti-rCD44v specific IgG antibody were measurable even at 1:12,800 dilution separately (Fig. 2a). Furthermore, titration of fecal and serum samples from immunized mice of T1 (oral), T2 (oral-injection), T3 (injection) and T4 (nano-injection) groups compared to control groups C1 (nano-injection), and C2 (oral) indicated a significant specific IgA response against rCD44v. And the higher titers of the specific IgA against rCD44v were enough high in both sera and fecal of all immunized mice groups even to compare with each other (Fig. 2b, c). After the last vaccination, the higher titers of anti-rCD44v IgG in the serum of all immunized groups were achieved.

### Neutralization assay

**Fig. 3:**
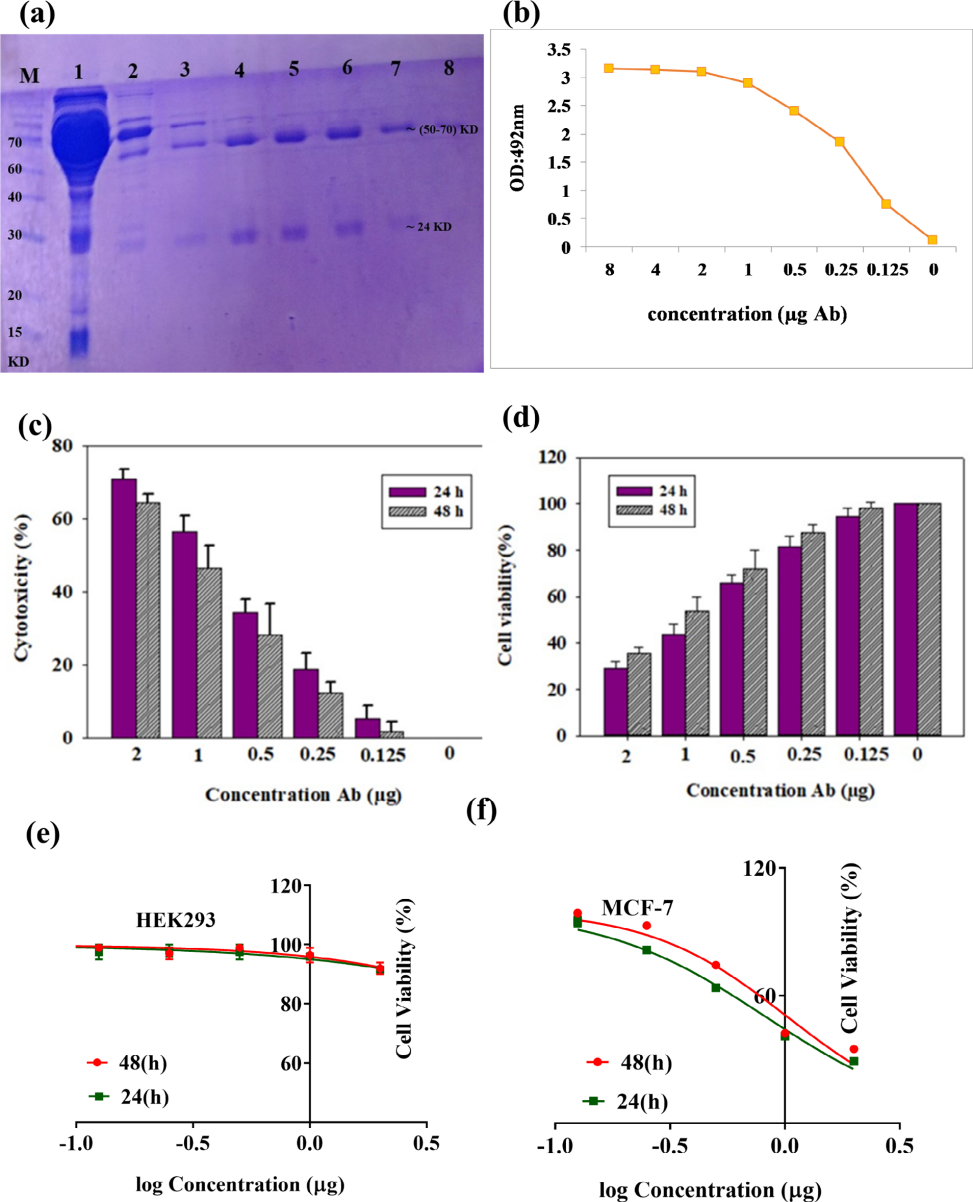
Purification of mouse anti-rCD44v antibodies in SDS PAGE gel (12%). Lane M, molecular weight marker. Lane 1, flow through. Lane 2, wash solution. Lanes 3, and 8, Elution buffer solution containing light and heavy chain bands of purified antibodies (about 24 and 50–70 KD) respectively (**a**). A spectrum of the purified antibody concentrations (8 to 0.125 µg) showed a very high absorbance value in ELISA with constant concentration (500 ng) recombinant CD44v antigen (**b**). Cytotoxic effects of the mouse anti-rCD44v on human breast cancer cells (MCF7 cell line) (**c**). Cell viability was assessed after 24 and 48 h of incubation with different concentrations (2 to 0.125 µg)by MTT assay (**d**). IC_50_ graphs of the mouse anti-rCD44v in HEK293 (**e**) and MCF7cells (**f**). Data represent the means ± SD of three independent experiments

To verify the in vitro cytotoxicity the mouse anti-rCD44v antibody, human breast cancer MCF7 (CD44^**+**^ cell line), and human normal HEK293 (CD44^**−**^ cell line) were treated with a spectrum of the purified antibody concentrations (2 to 0.125 µg) based on the amount of absorptions ELISA test (Fig. 3a, b). Based on MTT results no significant cytotoxic effect was revealed on HEK293 cells after cell treatment with mouse anti-rCD44v antibody. Furthermore, the HEK293 cell viability was nearly 94%, indicating the excellent safety of the mouse anti-rCD44v antibody.

In the present study, the mouse anti-rCD44v antibody reduced the viability of human breast cancer MCF7 in a concentration-dependent manner (Fig. 3c, d). Moreover, combination-index (CI_50_) values for the mouse anti-rCD44v antibody against MCF7 cells for 24 and 48 h were determined (0.8263 and 1.026 µg mL), respectively (Fig. 3e, f). Thus, the mouse anti-rCD44v had more cytotoxicity against breast cancer cells compared to the normal HEK293 cells.

### Growth of 4T1-CD44 tumor cells in mice

**Fig. 4:**
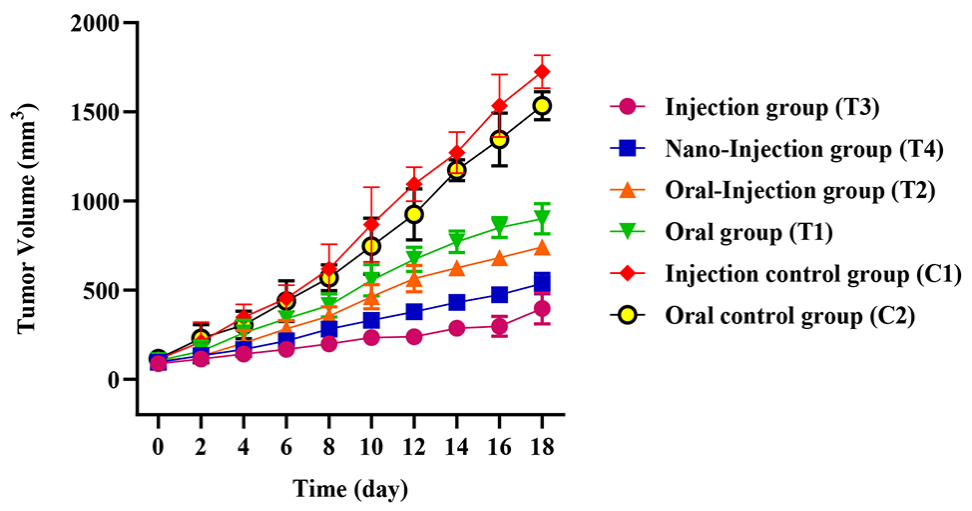
The changes in tumor volume following vaccination. The reduction of tumor growth was observed in the test groups BALB/c mice immunized with rCD44v as compared to the control group mice (*P* < 0.05)

Six groups of mice were challenged with 4T1-CD44 tumor cells and tumor volumes were assessed on day 14. The tumor dimensions were measured every 2 days (up to 20 days) to determine the association between elicited immune response following vaccination and the rate of tumor growth, [32]. In the comparisons of tumor sizes, it was revealed that tumor growth in the mice tests groups especially in injection and nano-injection compared with the mice control groups displayed a significant reduction (*P* < 0.05; Fig. 4).

### Antigen-specific cell proliferative responses

The potential of this vaccine to induce rCD44v-specific cell-mediated immune response was determined by in vitro cell proliferation assay after final tumor size measurement and the obtained results were considered as stimulation index (SI).

**Fig. 5:**
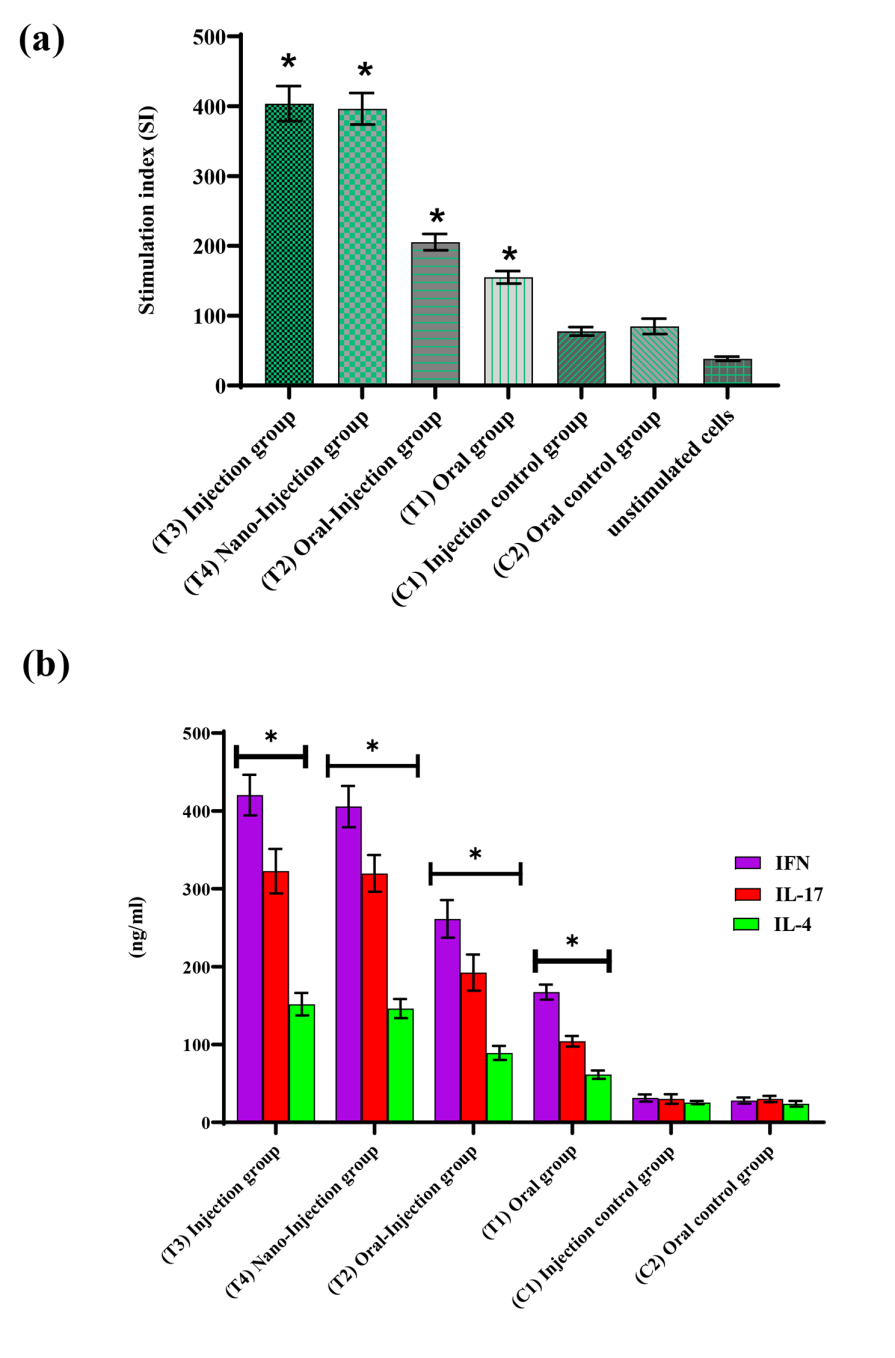
After the final tumor size measurement, splenocytes, collected from individual mice belonging to different experimental groups, were stimulated after 72 h of exposure to rCD44v (100 ng/µL), and the proliferative rates of mice lymphocyte cells were determined by MTT assay. Data represent the mean of stimulation index for 6 different mouse groups ± standard error. The difference in mean SI. Values between groups were assessed using ANOVA and comparisons were considered significant (Asterics show *p* < 0.05). Splenocytes were used without proximity to rCD44v (unstimulated cells) (**a**) Increase of IFNγ, IL-17, and IL-4 secretion by splenocytes isolated from BALB/c mice immunized with vaccine. Splenocytes were cultured in vitro with rCD44v (100ng/µL). After being cultured for 3 days, the supernatants were harvested, and IFNγ, IL-17, and IL-4 releases were measured by ELISA assay. The rCD44v showed significantly high levels of IFNγ and IL-17-producing ability in comparison to the control groups (Asterias show *P* < 0.05) (**b**). The mean ± SD of triplicate determinations is shown

As demonstrated in Fig. 5a, the vaccine significantly induced specific proliferation of splenocytes in immunized BALB/c mice in response to recombinant antigen in comparison with PBS or chitosan nanoparticles without antigens immunization (*P* < 0.05).

The rCD44v-triggered immune mechanism in splenocytes was evaluated through cytokines analyses. In all immunized BALB/c mice (including test (T1, T2, T3, T4) and control groups (C1, C2), The proportion of splenocytes secret IFNγ, IL-17, and IL-4 in response to in vitro rCD44v stimulation. The results are depicted in Fig. 5b.

A high amount of splenocytes secreting IFNγ and IL-17 was seen in immunized mice with rCD44v especially ( Injection and Nano-injection groups) (*P* < 0.05). Furthermore, a little higher amount of IL-4 secreting splenocytes was observed in immunized mice with the combined preparation compared to the PBS or chitosan nanoparticles without antigen mice control groups (*P* < 0.05). Our results illustrated that vaccination with rCD44v can induce Th1 type response towards CD44v.

### Histopathology, necrosis, and metastasis analyses of tumors

**Fig. 6:**
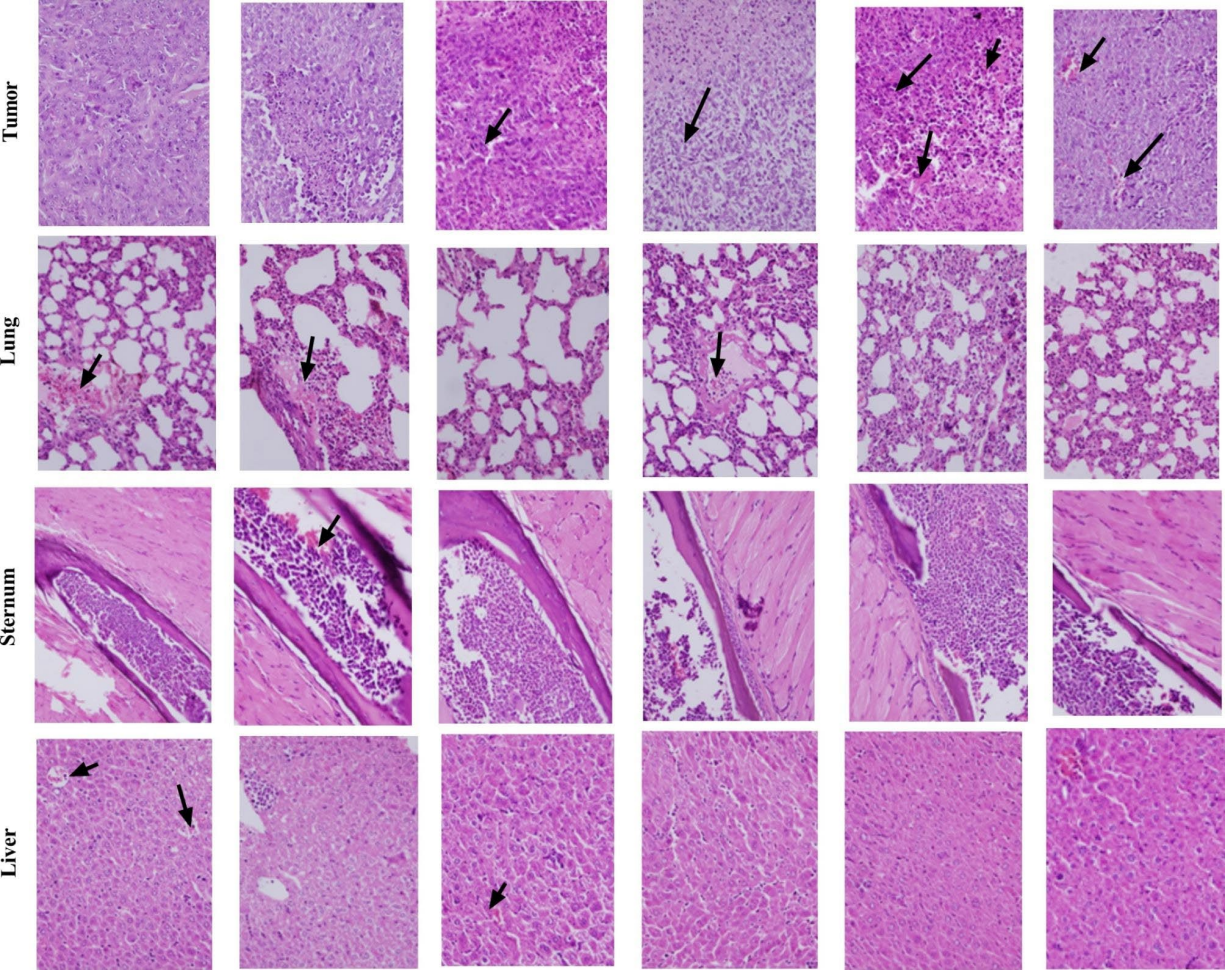
Histopathological observations of immunized mice organs (lung, liver, and sternum) and tumor tissues. Mice were immunized with PBS or rCD44v, Mice control (two groups), and Mice test (four groups). Tissue Sect. (3 μm) were stained with H&E and necrosis and metastases were examined by light microscopy. (40 × magnification and scale bar of 50 μm)

Histological examinations used H&E stained sections. The summary of which is illustrated in Fig. 6. Necrosis was significantly increased in the mice test groups (Injection and Nano-injection) (P < 0.05). Furthermore, mice vaccinated with rCD44v in the mice test groups (Oral and Oral-injection) clearly presented a smaller size of lung metastases compared to the control mice groups.

## Discussion

Primary human breast cancer contains a minority of the tumorigenic cell population (nearly 1–2% of the tumor mass) with a different phenotype from the other tumor cells and the capability of metastasis [33].

Since these specialized cells are the major cause of mortality in breast cancer patients, targeting these cells can be considered a useful potential to apply in the BCSC field. This could be accomplished by eliminating BCSCs via immunotherapy.

Identifying useful tumor antigens is one of the important strategies in immunotherapy which can effectively target tumor cells. Therefore, specific antigens with expression patterns limited to the cancer stem cells should be targeted [1].

Surface antigens are also known targets to design cancer vaccines. The most important characteristic of the tumor vaccines is specific response induction against the tumors, nontoxic effects, and prevention of recurrence of the tumor through long immunity [34, 35].

According to the review article presented by David Naor et al., malignant activities in a variety of neoplasms were markedly reduced by targeting CD44 through antibodies, antisense, and CD44-soluble proteins in animals, emphasizing the therapeutic potential of CD44 as a vaccine target. Therefore, the production of anti-CD44 agents specified for tumor cells can be considered a realistic therapeutic approach [3].

Anti-CD44 agents can also stimulate T cells and monocytes to trigger the secretion of cytokines, promoting the immune system for antitumor activity and inhibiting tumor development and metastasis [6, 36]. In an appropriate immunotherapy procedure, a strong induction of anti-tumor T-cell response is critical [34]. This can be achieved by designing a multimodal cancer vaccine to activate different arms of the immune system at once. Designing a vaccine to elicit both cellular and humoral immune responses seems difficult, although it has been observed through anti-CD44 vaccination in some cancer patients [1, 3].

Also, Prashant Kesharwani et al. reviewed the role of CD44 in tumorigenesis and its potential as a therapeutic target using several delivery nanocarriers such as polymeric/nonpolymeric nanoparticles, dendrimer, micelles, carbon nanotubes, nanogels, nanoemulsions, etc., for targeted delivery of several chemotherapeutic molecules and nucleic acid [37].

To address these issues, chitosan can stimulate cellular immune responses, presenting a proper candidate for the cancer vaccine as an adjuvant. It can also elevate the maturity of the dendritic cells [38].

In this study, encapsulated rCD44v protein with chitosan nanoparticles was used due to different reasons. First, for better delivery of the vaccine to the target cells. Second, to protect the rCD44v protein from digestion and degradation. And finally, to elicit immunogenicity of the designed vaccine. Such kinds of vaccines are benefited from improved immunogenicity and ease of production compared to the traditional vaccine [39].

Based on our nano-vaccine physical analyses (the particle size and Zeta potential), the results of EE% and antigen-releasing from nanoparticles confirmed a steady release of the rCD44v from the surface of the nanoparticle followed by a gradual and suitable stimulation of the immune system [40, 41].

It has been shown that the accepted Zeta potential range should lie between − 20 mV and + 30 mV. In this study, we found the Zeta potential of chitosan nanoparticles nearly + 3.3 ± 0.16 (Mean ± SD) that indicated the formation of a stable formulation. Generally, the carboxyl group of chitosan is responsible for positive Zeta potential values of chitosan nanoparticles [42]. In nanosuspensions, chemical stability is a minor concern; however, this issue is considerable when the solubility of drug molecules is greater than 1 mg/mL [19].

There are no significant differences between CD44 gene sequences in humans and mice [3, 43], and our human-based CD44 vaccine could be applied in mice, as an appropriate immunological model, to elucidate its potential immunotherapeutic effect. Therefore, we recruited a mouse model for breast cancer to validate the immunization activity of our designed rCD44v vaccine and found that rCD44v efficiently reduced both tumor growth and metastases.

Based on the ELISA results, anti-CD44v antibody was raised in all immunized mice groups. The IgG titers were higher in the rCD44v immunized mice compared to the control groups, following oral injection and oral vaccine administration. We found here that the 4th round of vaccine injection was critical to obtain an increased titer of IgG antibodies in the oral- injection group compared to the oral group, probably due to considerable difference in introduced IgG antibodies titer. Instead, the titer of IgA antibody was elevated in the oral than oral-injection group, probably due tomore antigen delivery at mucosal surfaces. Two reasons may be involved in this IgA titer elevation in the oral group; the interaction between negatively charged mucosal surfaces and positively charged nanoparticles, and the suitable adhesion of nanoparticles to mucosal surfaces [9, 44]. While IgA titration was lower in injection administration in comparison with other groups, IgG titration was higher in our study (Fig. 2).

MTT test was used to examine the cell proliferation of mouse splenocytes. A greater proliferation of the cells leads to an increase in SI. Based on our results, rCD44v caused a better stimulation and proliferation of test groups’ splenocytes compared to controls (Fig. 5). Furthermore, rCD44v stimulated splenocytes to produce cytotoxic cytokines (INFγ) demonstrating its important function in necrosis and tumor cell lysis. The mechanism of necrosis induction is not well understood, however, Cytotoxic T lymphocytes (CTLs) and the secreted cytokines may be involved in triggering injuries.

Figures 4 and 5b describe the tumor size and cytokines analysis, respectively. Figure 6 illustrates the tumor necrosis and metastases. As can be seen in Fig. 4, tumor growth is significantly decreased in injection and nano-injection mice compared to control groups (*P* < 0.05). Additionally, the titers of IL-17, IFNγ, and IL-4 were meaningfully different in tests mice in comparison with control groups (*P <* 0.05; Fig. 5b). Based on the level of IFNγ secretion, we revealed that the rCD44v induced a Th1 response toward the designed recombinant protein, while the Th2 response was rather weak. Thus, the elevated levels of IL-17 and IFNγ compared to IL-4 show that the recombinant CD44v protein boosts the antitumor activity. Our findings demonstrated that the size of tumor necrosis and metastases was significantly different in the test mice compared to the control groups (*P <* 0.05), following rCD44v administration. These findings are in line with our results in tumor size measurement, probably confirming the potential of anti-rCD44v as an efficient cancer vaccine to inhibit tumor growth.

Also, the neutralization assay results showed that mouse polyclonal antibodies were directly cytotoxic on human cancer cells (CD44^+^) about 70%, but did not affect human normal cells (CD44^**−**^). Our elucidation of rCD44v potential as a cancer vaccine may pave the road for its use in the clinical management of breast cancer patients. This in vivo study revealed that rCD44v can be useful to induce antitumor effects. Although the introduced methods in this study can be used instead of routine adjuvants, it is expensive. Further detailed investigations are needed to evaluate its probable side effects.

## Conclusion

In conclusion, we approved that our newly designed construct significantly induced both humoral and cellular immune responses in vivo. Since the CD44v antigenicity differs between malignant and normal tissues, our results confirmed that rCD44v chitosan-based cancer vaccine can effectively function against tumor growth and metastasis, and can be considered a great promise as well as highly valuable to use in medical oncology.

## Electronic supplementary material

Below is the link to the electronic supplementary material.

Supplementary Material 1

Supplementary Material 2

Supplementary Material 3

## Data Availability

All raw data are available in case of a reasonable request from the corresponding author.

## Abbreviations

CSCs: cancer stem cells.
BCSCs: breast cancer stem cells.
TAAs: tumor-associated antigens.
CD44: Cluster of Differentiation 44.
CD44v: CD44 variable isoforms.
rCD44v: recombinant CD44 variable isoforms.
CFA: Complete Freund’s adjuvant.
IFA: incomplete Freund adjuvant.
DMEM: Dulbecco’s modified Eagle’s medium.
DMSO: dimethyl sulfoxide.
PBS: phosphate-buffered saline.
PBST: phosphate-buffered saline containing 0.05% Tween-20.
MTT: 3-(4,5-dimethylthiazol-2-yl)-2,5-diphenyltetrazolium bromide.
SI: stimulation index.
SD: standard deviation.
ELISA: enzyme-linked immunosorbent assay.
BSA: Bovine serum albumin.
IC_50_: half-maximal inhibitory concentration.
H&E: hematoxylin and eosin.
OPD: O-phenylenediamine.
TPP: tripolyphosphate.
DLS: dynamic light scattering.
OD: optical intensity.
PDI: polydispersity index.
CTLs: Cytotoxic T lymphocytes.
IFN-γ: Interferon gamma.
IL-17: Interleukin-17.
IL-4: Interleukin-4.
