# Supplementary figures and images for: Preparation of chitosan nanoparticle containing recombinant CD44v antigen and evaluation of its immunization capacity against breast cancer in BALB/c mice
Source: BMC Cancer. 2023 Feb 9;23:134. doi: 10.1186/s12885-023-10614-x (PMC9912563; doi:10.1186/s12885-023-10614-x)

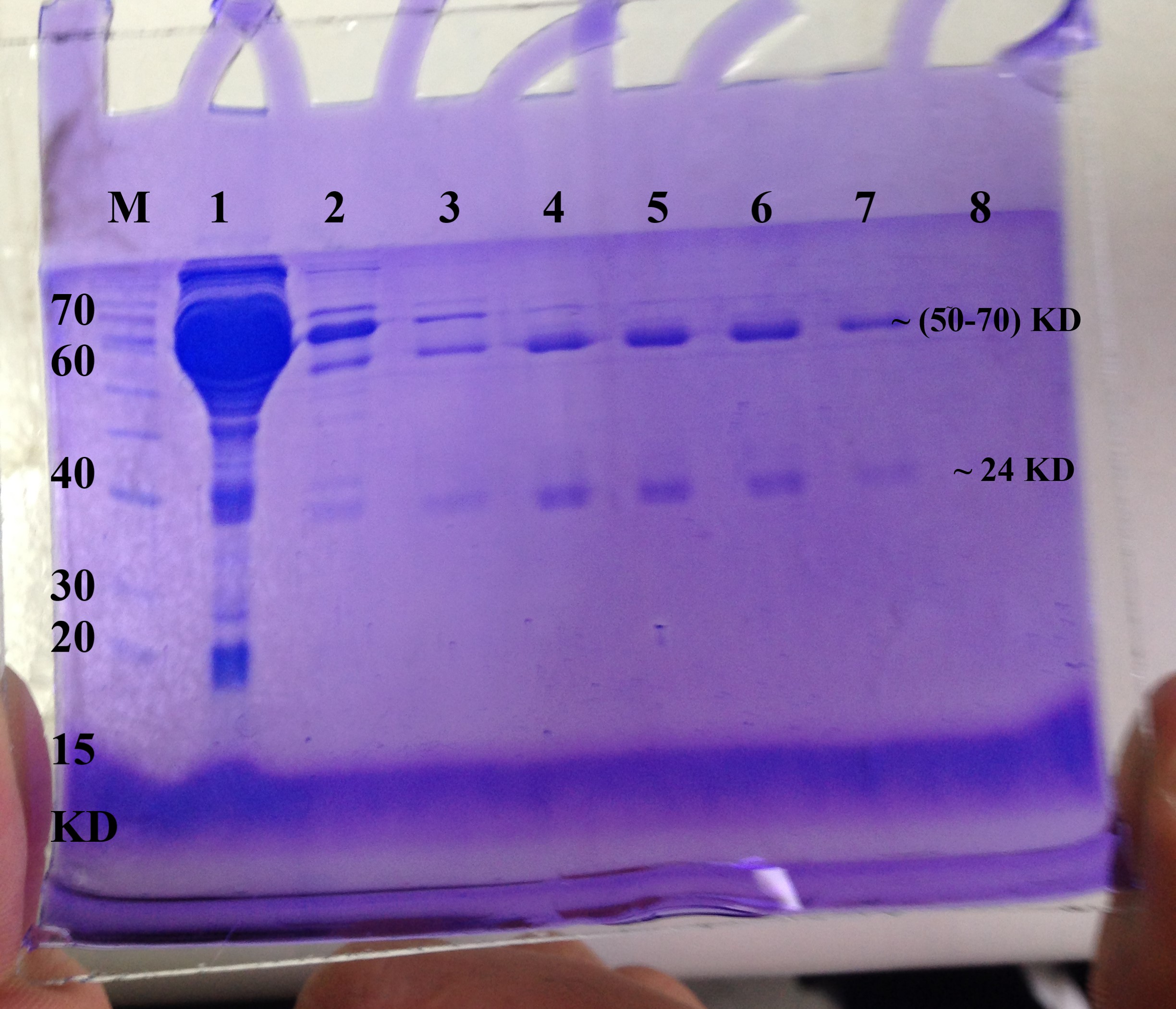

Supplement: Supplementary file 1 — Supplementary Material 1 [file 12885_2023_10614_MOESM1_ESM.jpg]

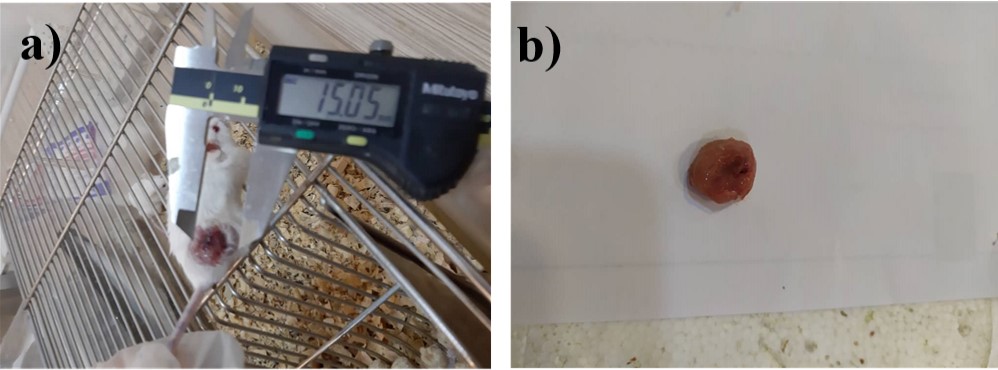

Supplement: Supplementary file 2 — Supplementary Material 2 [file 12885_2023_10614_MOESM2_ESM.jpg]
