# Supplementary material for: Preparation of chitosan nanoparticle containing recombinant CD44v antigen and evaluation of its immunization capacity against breast cancer in BALB/c mice
Source: BMC Cancer. 2023 Feb 9;23:134. doi: 10.1186/s12885-023-10614-x (PMC9912563; doi:10.1186/s12885-023-10614-x)
Supplement: Supplementary file 3 — Supplementary Material 3 [file 12885_2023_10614_MOESM3_ESM.docx]

**Supplementary figure legends**

**Supplementary Figure 1**. Purification of mouse anti-rCD44v antibodies in SDS PAGE gel (12%). Lane M, molecular weight marker. Lane 1, flow through. Lane 2, wash solution. Lanes 3, and 8, Elution buffer solution containing light and heavy chain bands of purified antibodies (about 24 and 50-70 kD), respectively.

**Supplementary Figure 2. a)** The tumor growth was measured using a digital caliper in the mice. **b)** Isolated tumor from the mice for further examination.
